# Development of a Stable Respiratory Syncytial Virus Pre-Fusion Protein Powder Suitable for a Core-Shell Implant with a Delayed Release in Mice: A Proof of Concept Study

**DOI:** 10.3390/pharmaceutics11100510

**Published:** 2019-10-03

**Authors:** Max Beugeling, Katie Amssoms, Freek Cox, Ben De Clerck, Ellen Van Gulck, Jeroen A. Verwoerd, Guenter Kraus, Dirk Roymans, Lieven Baert, Henderik W. Frijlink, Wouter L. J. Hinrichs

**Affiliations:** 1 Department of Pharmaceutical Technology and Biopharmacy, University of Groningen, Antonius Deusinglaan 1, 9713 AV Groningen, The Netherlands; m.beugeling@rug.nl (M.B.); jeroen_verwoerd@hotmail.com (J.A.V.); h.w.frijlink@rug.nl (H.W.F.); 2 Drug Product Development_Developability, Janssen Research and Development, a Division of Janssen Pharmaceutica NV, Turnhoutseweg 30, 2340 Beerse, Belgium; kamssoms@its.jnj.com; 3 Janssen Vaccines & Prevention B.V., Archimedesweg 4-6, 2333 CN Leiden, The Netherlands; FCox@its.jnj.com; 4 Janssen Infectious Diseases and Vaccines, Janssen Research and Development, a Division of Janssen Pharmaceutica NV, Turnhoutseweg 30, 2340 Beerse, Belgium; BDECLERC@its.jnj.com (B.D.C.); EVANGULC@its.jnj.com (E.V.G.); gkraus@its.jnj.com (G.K.); DROYMANS@its.jnj.com (D.R.); 5 Jalima Pharma bvba, Jozef Van Walleghemstraat 11, 8200 Brugge, Belgium; lieven.baert@jalimapharma.com

**Keywords:** biphasic pulsatile release, controlled release, freeze-dried powder, fusion protein, poly(dl-lactic-*co*-glycolic acid), pre-fusion, respiratory syncytial virus, single-injection vaccine

## Abstract

Currently, there is an increasing interest to apply pre-fusion (pre-F) protein of respiratory syncytial virus (RSV) as antigen for the development of a subunit vaccine. A pre-F-containing powder would increase the flexibility regarding the route of administration. For instance, a pre-F-containing powder could be incorporated into a single-injection system releasing a primer, and after a lag time, a booster. The most challenging aspect, obtaining the booster after a lag time, may be achieved by incorporating the powder into a core encapsulated by a nonporous poly(dl-lactic-*co*-glycolic acid) (PLGA) shell. We intended to develop a stable freeze-dried pre-F-containing powder. Furthermore, we investigated whether incorporation of this powder into the core-shell implant was feasible and whether this system would induce a delayed RSV virus-neutralizing antibody (VNA) response in mice. The developed pre-F-containing powder, consisting of pre-F in a matrix of inulin, HEPES, sodium chloride, and Tween 80, was stable during freeze-drying and storage for at least 28 days at 60 °C. Incorporation of this powder into the core-shell implant was feasible and the core-shell production process did not affect the stability of pre-F. An in vitro release study showed that pre-F was incompletely released from the core-shell implant after a lag time of 4 weeks. The incomplete release may be the result of pre-F instability within the core-shell implant during the lag time and requires further research. Mice subcutaneously immunized with a pre-F-containing core-shell implant showed a delayed RSV VNA response that corresponded with pre-F release from the core-shell implant after a lag time of approximately 4 weeks. Moreover, pre-F-containing core-shell implants were able to boost RSV VNA titers of primed mice after a lag time of 4 weeks. These findings could contribute to the development of a single-injection pre-F-based vaccine containing a primer and a booster.

## 1. Introduction

Respiratory syncytial virus (RSV) is a major cause of acute lower respiratory infections in infants and young children [1,2]. Globally, the incidence of acute lower respiratory infections caused by RSV was approximately 33.1 million in children younger than 5 years in 2015. Around 3.2 million children required hospital admission and the mortality was estimated to be 118,200 [3]. In addition, the virus causes serious disease in certain high-risk populations, such as the elderly and adults with a chronic heart or lung disease [4]. Despite the significant global health burden caused by RSV, no safe and efficient vaccine is available. In 1966, an attempt was made with a formalin-inactivated RSV vaccine meant for infants and young children [5,6]. However, this attempt failed and resulted in the death of two young children [6]. The failed trial shifted research toward the development of a subunit-based RSV vaccine meant for the elderly and pregnant women (to passively vaccinate infants).

Subunit-based vaccines consist of specific antigens that are able to elicit an immune response [7,8]. For RSV subunit-based vaccines, there is a growing interest in the application of the pre-fusion (pre-F) protein as antigen. This protein is a highly conserved trimeric membrane protein that mediates fusion with host cells [9,10]. The pre-F protein may flip into the post-fusion (post-F) conformation, which is known to less effectively induce RSV neutralizing responses than pre-F [9,11,12]. Therefore, it is important for a pre-F-based RSV subunit vaccine to maintain the pre-F conformation during isolation, formulation, and storage.

In the development of subunit-based vaccine products, often only liquid vaccine formulations for parenteral administration are considered, such as the liquid pre-F vaccine candidate described by Krarup et al. [13]. A pre-F-containing powder, however, would increase the flexibility regarding the route of administration of the vaccine. Such a powder could be incorporated into several other dosage forms, such as sublingual tablets [14], microneedles, powder for intranasal or pulmonary administration, or solid-state-controlled release devices [15].

However, a major consideration to take into account in the development of a dry powder is that the drying process itself may be destructive for the protein and may therefore lead to a decrease, or even a total loss, of activity [16]. To stabilize proteins during drying, the use of several excipients (e.g., sugars, buffers, surfactants, and salts) has been described [17,18]. Moreover, it is known from literature that sugars not only stabilize proteins during drying, but also during storage [16,19]. Another advantage of the use of excipients during drying is that they can serve as bulking agents [20], making it possible to accurately dose small amounts of the antigen and incorporate it into more complex dosage forms.

Many subunit-based vaccines, potentially including a pre-F-based subunit vaccine, are poorly immunogenic [7,8]. Therefore, they often require multiple injections to achieve the desired immune response able to protect the vaccinee [7,8]. Following the first administration (primer), one or more subsequent administrations (booster) of the vaccine are necessary to achieve the desired immunological reaction [21,22]. Such a multiple-injection regime is disadvantageous because it might endanger compliance and thus vaccination efficacy [23,24]. To overcome this, a single-injection system containing a primer and a booster dose of pre-F could be developed. Such a device should release the primer immediately, while the booster is released after a certain lag time as a pulse, also known as a biphasic pulsatile release profile [23,24,25]. Obtaining the booster is the most challenging aspect of such a system, as the release of the antigen should occur after a certain lag time following administration, during which the antigen is exposed to an elevated temperature, i.e., body temperature.

Recently, we have shown that core-shell implants consisting of a solid state ovalbumin (OVA)-containing core encapsulated by a nonporous poly(dl-lactic-*co*-glycolic acid) (PLGA) shell could induce a delayed IgG1 antibody response in mice [26]. To mimic a single-injection vaccine containing a primer and a booster dose, mice were primed with a subcutaneous (s.c.) injection of OVA, as the primer was not yet included in the device [26]. The PLGA shell around the OVA-containing core was nonporous and thereby acted as a barrier, preventing continuous OVA release. However, after a lag time of approximately 3–4 weeks, the PLGA was degraded to such an extent that it could no longer serve as a barrier, resulting in the release of OVA [26].

To increase the flexibility regarding the route of administration, we intended to develop a stable RSV pre-F protein powder formulation and drying process. In addition, we investigated whether incorporation of this powder into the core-shell implant was feasible and whether this system could induce a delayed RSV virus-neutralizing antibody (VNA) response in mice.

## 2. Materials and Methods

### 2.1. Materials

Trimeric RSV pre-F protein (3.38 mg/mL (based on UV 280 nm) in 40 mM Tris buffer pH 7.5 with 150 mM NaCl), comparable to the vaccine candidate described by Krarup et al. [13], was produced by U-Protein Express BV (Utrecht, The Netherlands) on request of the authors. The purity of the protein was >96% and the molecular weight of the protein was approximately 175 kDa (confirmed with non-reducing SDS-PAGE and size-exclusion chromatography). Inulin with a degree of polymerization of 23 was obtained from Sensus (Roosendaal, The Netherlands). Di-sodium hydrogen phosphate dihydrate, sodium dihydrogen phosphate monohydrate, sodium chloride, sodium hydroxide, and sulfuric acid were purchased from Merck KGaA (Darmstadt, Germany). Monopotassium phosphate and potassium chloride were purchased from BUFA (IJsselstein, The Netherlands). Sodium phosphate dibasic, Tween 20, Tween 80, bovine serum albumin (BSA), Tris base, 8-anilino-1-napthalenesulfonic acid ammonium salt (ANS), 4-(2-hydroxyethyl)-1-piperazineethanesulfonic acid (HEPES), and l-leucine were purchased from Sigma-Aldrich Co. (St. Louis, MO, USA). Pearlitol 200 SD (mannitol) was purchased from Roquette (Roosendaal, The Netherlands). CR9506 (3.66 mg/mL), CR9501-biotin (9.91 mg/mL), CR9506-biotin (3.10 mg/mL), and RSV CL57V224 labeled with firefly luciferase (RSV CL57V224-FFL) were obtained from Janssen Infectious Diseases and Vaccines (Leiden, The Netherlands). Streptavidin horseradish peroxidase (strep-HRP) was purchased from BD Biosciences (Franklin Lakes, NJ, USA). *O*-phenylenediamine dihydrochloride (OPD) tablets (5 mg/tablet), 10× stable peroxide substrate buffer, and phosphate-buffered saline (PBS; pH 7.2) were purchased from Thermo Fisher Scientific (Waltham, MA, USA). PLGA with a lactic:glycolic acid ratio of 50:50 and an intrinsic viscosity of 0.2 dL/g (PURASORB^®^PDLG 5002) was purchased from Corbion Purac Biomaterials (Amsterdam, The Netherlands). Blood collection tubes were from Sarstedt AG & Co (Nürnbrecht, Germany). Neolite substrate was purchased from PerkinElmer (Waltham, MA, USA). Water used in all experiments was Millipore, type 1 water.

### 2.2. Production of the Freeze-Dried Powders

First, an inulin solution was prepared by dissolving 1.75 g inulin in 28.4 mL Millipore water. This was done in a closed container under continuous heating on a hot plate (80 °C). The solution was allowed to cool down to room temperature (RT) after the inulin was completely dissolved. An amount of 87.2 mg sodium chloride was added to the cooled solution. Subsequently, an amount of 700 μL of a 1 M HEPES buffer solution (pH 7.5) and 700 μL of a 1% (*v*/*v*) Tween 80 solution were added. Finally, 5.23 mL of the aqueous pre-F stock solution (3.38 mg/mL) was added to the solution, resulting in a formulation containing pre-F:inulin 1:99 (*w*/*w*), 20 mM HEPES (pH 7.5), 65 mM NaCl, and 0.02% (*v*/*v*) Tween 80. A placebo powder containing all excipients was made similarly.

Samples of 200 μL (containing approximately 101 μg pre-F) in 2-mL Eppendorf tubes and samples of 2 mL in 10 mL injection vials were rapidly frozen by immersing the samples in liquid nitrogen. The samples were immediately placed on the shelf of the freeze-dryer (Christ Epsilon 2-4 LSCplus, Salm & Kipp, Breukelen, The Netherlands), which was pre-cooled to a temperature of −35 °C. The primary drying step was for 32 h at a pressure of 0.220 mBar. The secondary drying step was for 12 h at a pressure of 0.055 mBar, while the temperature was gradually increased to RT. The obtained dry powder was handled under dry nitrogen gas and the Eppendorf tubes were vacuum-packed with a sealing machine (Dinamika 570, Solis, Opfikon, Switzerland). The injection vials were hermetically sealed.

### 2.3. Storage Stability of the Freeze-Dried Pre-F-Containing Powder

The storage stability of the freeze-dried pre-F-containing powder was investigated by placing the vacuum-packed Eppendorf tubes containing approximately 101 μg of pre-F in ovens at 37 °C (to mimic body temperature) and 60 °C (to mimic long-term stability) (Termaks TS 8265, Bergen, Norway). At predetermined time points (*t* = 0, 7, 14, 21, and 28 days), samples were analysed using enzyme-linked immunosorbent assay (ELISA), size-exclusion chromatography (SEC), and fluorescence spectroscopy.

### 2.4. Enzyme-Linked Immunosorbent Assay (ELISA)

Samples of the freeze-dried pre-F powder containing approximately 101 μg pre-F (*n* = 3) were reconstituted in 2 mL PBS (pH 7.4) and diluted appropriately with sample buffer (PBS supplemented with 0.1% (*w*/*v*) BSA and 0.05% (*v*/*v*) Tween 20). A calibration curve with a range of 1.875–40 ng/mL was made with unprocessed pre-F diluted in sample buffer.

A method similar to the one described by Krarup et al. [13] was used. First, the capturing antibody, CR9506, was diluted in PBS to a concentration of 1 μg/mL. Of this solution, 100 μL was added to the wells of a 96-well Nunc MaxiSorp plate (Thermo Fisher Scientific, Waltham, MA, USA). The plate was incubated overnight at a temperature of 4 °C. The plate was washed three times with PBS supplemented with 0.05% (*v*/*v*) Tween 20 (PBST), after which 200 μL blocking buffer (PBS containing 1% BSA (*w*/*v*)) was added to the wells. The plate was incubated for 1 h at RT. After incubation, the plate was washed three times with PBST. Then, 100 μL sample was added to each well. Samples of the reconstituted freeze-dried powder were added in duplicate. After 1 h of incubation at RT, the plate was washed again three times with PBST. An amount of 100 μL of either CR9501-biotin (binds specifically to the pre-F conformation of the F protein) or CR9506-biotin (binds to both, the pre-F conformation and the post-F conformation of the F protein), both diluted with sample buffer to a concentration of 0.25 μg/mL, was added to the wells. After 1 h of incubation at RT, the plate was washed again three times with PBST. Subsequently, 100 μL strep-HRP, diluted 1:1,000 (*v*/*v*) with sample buffer, was added to each well and the plates were incubated in the dark for 1 h. Ten minutes before washing the plate for the last time, two OPD tablets were dissolved in 18 mL Millipore water. When the tablets were completely dissolved, 2 mL of 10× stable peroxide substrate buffer was added. The plate was washed three times with PBST and 100 μL of the OPD solution was added to each well. Color was allowed to develop in the dark for 15 min for the plate with CR9501-biotin as detection antibody, and 20 min for the plate with CR9506-biotin as detection antibody. Thereafter, 100 μL of a 1 M H_2_SO_4_ solution was added to each well to stop the reaction. The plate was read at a wavelength of 492 nm using a Biotek Synergy HT multi-detection microplate reader (Winooski, VT, USA). Data integration was performed by using GraphPad Prism 6.0c.

### 2.5. Size-Exclusion Chromatography (SEC)

SEC was performed to investigate the presence of irreversible soluble aggregates and/or fragments and to quantify trimeric pre-F. The experiments were performed using a Dionex UltiMate 3000 RS HPLC system (Thermo Fisher Scientific, Waltham, MA, USA) and the corresponding Chromeleon software. The guard column used was a WTC-050S5G SEC guard column and the column was a WTC-05S5 SEC protein column (Wyatt, Santa Barbara, CA, USA). As running buffer, a 0.2 μm filtered 0.2 M phosphate buffer (PB; pH 6.8) was used. The flow rate was set at 0.8 mL/min. During the experiments, the temperature of the autosampler was set at 4 °C, while the temperature of the column compartment was set at 20 °C. An injection volume of 40 μL was used and the detector was set at a wavelength of 280 nm. The run time was set at 26 min.

In order to quantify trimeric pre-F, a calibration curve of unprocessed pre-F diluted in PB (pH 6.8) with a range of 15.625–500 μg/mL was made in triplicate. Samples of the freeze-dried pre-F powder containing approximately 101 μg pre-F (*n* = 3) and samples of the freeze-dried placebo powder (*n* = 3) were reconstituted in 800 μL running buffer. All samples were filtered through 0.2 μm filters. By integrating the peaks and using the calibration curve, the recovery (% trimeric pre-F of theoretical) was calculated. Moreover, the chromatograms were evaluated for the existence of additional peaks.

### 2.6. Fluorescence Spectroscopy

Intrinsic and extrinsic fluorescence spectroscopy methods similar to the ones described by Amssoms et al. [26] were used to investigate whether pre-F maintained its native conformation. For these experiments, a fluorospectrometer (Quantamaster 40, Photon Technology International, Inc., Birmingham, NJ, USA) and the corresponding FelixGX software was used. The slit width was set at 2.5 nm and the temperature of the sample holder was set at 20 °C. Samples of the freeze-dried pre-F powder containing approximately 101 μg pre-F (*n* = 3) and samples of the freeze-dried placebo powder (*n* = 3) were reconstituted in 2 mL PBS (pH 7.4). Fresh unprocessed pre-F samples (*n* = 3) diluted in PBS (pH 7.4) with the same concentration as the reconstituted samples were prepared as controls. All samples were filtered through 0.2 μm filters. Intrinsic fluorescence spectroscopy and extrinsic fluorescence spectroscopy were performed and the background signal was subtracted from the signal of the samples containing pre-F.

#### 2.6.1. Intrinsic Fluorescence Spectroscopy

For intrinsic fluorescence spectroscopy, a fluorescence Quartz cuvette (*L* = 10 mm, Hellma GmbH & Co. KG, Müllheim, Germany) was filled with 1.5 mL sample. The cuvette containing the sample was placed in the sample holder of the fluorospectrometer and the sample was constantly stirred during the measurement. The samples were excited at a wavelength of 295 nm and an emission spectrum was taken from 300 to 360 nm.

#### 2.6.2. Extrinsic Fluorescence Spectroscopy

The extrinsic fluorescence was measured immediately after the intrinsic fluorescence of the sample. In order to do this, 18.8 μL of a 2 mM ANS solution was added to the sample. In addition, unprocessed pre-F samples (*n* = 3) diluted in PBS (pH 7.4) with the same concentration as the reconstituted freeze-dried pre-F-containing samples were exposed to a heat shock for 60 min at 60 °C (HS pre-F 60 min 60 °C) and a heat shock for 15 min at 100 °C (HS pre-F 15 min 100 °C) as a negative controls. All samples were excited at a wavelength of 386 nm and an emission spectrum was taken from 400 to 600 nm.

### 2.7. Production of Pre-F-Containing Cores

Pre-F-containing oblong core tablets with dimensions of 6 × 2 mm were produced using the freeze-dried pre-F-containing powder. The core consisted of a physical mixture of the freeze-dried pre-F-containing powder (24 wt %) and mannitol (76 wt %). First, the freeze-dried powder was ground and mixed with mannitol in a smooth agate mortar with a pestle for 5 min. Cores of 25 mg (containing 50 μg pre-F) were produced from this powder mixture by using a compaction apparatus (Instron 5960, Norwood, MA, USA) at a compaction load of 3 kN and a compaction rate of 0.5 kN/s. The pressure was ramped up linearly over 6 s, held for 0.1 s, and then ramped down over 6 s. l-leucine was used as an external lubricant. The produced cores were stored under dry nitrogen gas in Eppendorf tubes, and vacuum-packed using a sealing machine. Placebo cores containing 24 wt % freeze-dried placebo powder and 76 wt % mannitol were produced in the same way.

### 2.8. Production of Core-Shell Implants

**Figure 1:**
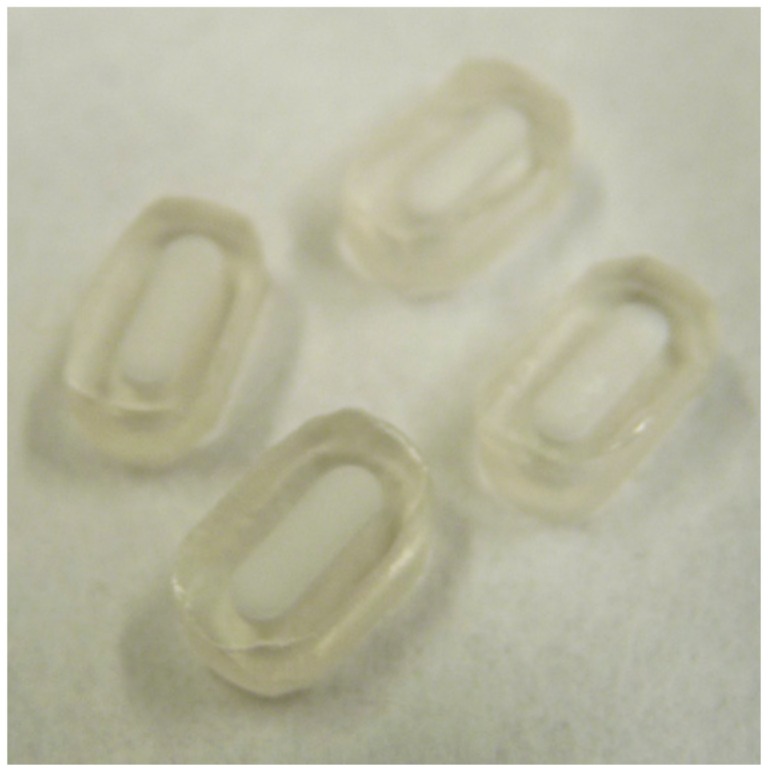
Photograph (top view) of four oblong pre-F-containing core-shell implants (approximately 5 × 9 mm). The core (approximately 25 mg) consisted of a physical mixture of freeze-dried pre-F-containing powder (24 wt %) and mannitol (76 wt %). The shell consisted of PLGA.

The core-shell implants were produced similarly to the OVA-containing core-shell implants previously described by Amssoms et al. [26]. The particle size of the polymer was first reduced by grinding PLGA three times for approximately 5 s with a grinder (Moulinex AR100, Écully, France), resulting in a polydisperse mixture of PLGA particles (particle sizes ranging from approximately 1 mm to approximately a few μm). To produce the PLGA shell around the pre-F-containing core, an amount of 125 mg PLGA was vacuum-compressed using a preheated (48 °C) tablet die with a diameter of 9 mm. A temperature of 48 °C was applied because this temperature is well above the glass transition temperature (Tg) of the polymer (inflection point of approximately 36.5 °C), allowing for sufficient viscous flow to obtain a nonporous shell. Compaction was done at a compaction load of 5 kN and a compaction rate of 0.5 kN/s. The pressure was ramped up linearly over 10 s, held for 10 s, and then ramped down over 10 s. The pre-F-containing core was placed in the middle of the PLGA containing die, and another 125 mg PLGA was added as top layer and vacuum-compressed using the same settings (compaction load of 5 kN, compaction rate of 0.5 kN/s, and a hold time of 10 s). Additional heating for 20 min at 48 °C was applied by putting the die containing the core-shell implant in an oven. To close any pores, the core-shell implant was re-compressed at a compaction load of 0.5 kN, a compaction rate of 0.5 kN/s, and a hold time of 10 s. Before removing the core-shell implant from the tablet die, the tablet die was allowed to cool down to a temperature below 40 °C. The core-shell implants were reduced to an oblong shape with a size of approximately 5 × 9 mm by using a multi-tool (Dremel 4000, Racine, WI, USA). Figure 1 shows a photograph (top view) of four pre-F-containing core-shell implants. Core-shell implants containing a placebo core were produced in the same way.

### 2.9. Influence of the Core-Shell Production Process on the Stability of Pre-F

To investigate whether the core-shell production process affects the stability of pre-F, pre-F-containing core-shell implants were analysed using ELISA, SEC, and fluorescence spectroscopy (methods described above). For each analytical technique, pre-F-containing core-shell implants (*n* = 3) were first cut into four pieces using a surgical blade in order to expose the pre-F-containing core. Subsequently, the pieces were transferred to Eppendorf tubes and the cores were dissolved in the appropriate medium. If necessary, the samples were further diluted. Fresh unprocessed pre-F samples (*n* = 3) with the same theoretical concentration as the pre-F-containing core-shell implants were prepared as controls for fluorescence spectroscopy.

### 2.10. In Vitro Release Study with Pre-F-Containing Core-Shell Implants

An in vitro release study was performed to investigate the release of pre-F from the pre-F-containing core-shell implants. Pre-F-containing core-shell implants were placed in closable injection vials (one per vial, *n* = 3), containing 20 mL of 100 mM PBS (pH 7.4, supplemented with 0.02% (*w*/*v*) sodium azide) as release medium. Subsequently, the vials containing the core-shell implants were placed in a shaking water bath (80 rpm) at 37 °C. At predetermined time points, 18 mL of the release medium was sampled and 18 mL of fresh preheated (37 °C) release medium was pipetted back into the vial. Before taking the samples, the solutions were homogenized. The release samples were diluted appropriately and analysed using ELISA (method described above). ELISA was chosen to monitor the release of pre-F because this technique is highly sensitive and makes it possible to investigate the conformational integrity of the released pre-F.

### 2.11. Animals

Female 8–12-week-old BALB/c mice, weighing 20–25 g, were obtained from Javier (Le Genest-Saint-Isle, France). The animals were housed in individually ventilated cage racks and were provided with food and water *ad libitum*, in accordance with the institutional and national guidelines. All in vivo experiments were conducted under approval from the Janssen Ethics Committee on Animal Experiments (Permit number: ‘Proj 019 OVA’, date of approval: 4 May 2017).

### 2.12. Mouse Immunization and Sample Collection

Twenty-seven mice were divided into 5 groups: the treatment groups (groups A and B), the positive control group (group C), and the placebo group (group D), each contained 6 mice. The negative control group (group E) contained 3 mice. In Table 1, an overview of the experimental groups and the corresponding formulations used for the in vivo study is given. Mice of group A were immunized s.c. with the pre-F-containing core-shell implant on day 0. Mice of group B were immunized s.c. with a prime vaccination of pre-F (50 μg) diluted in PBS solution (200 μL; pH 7.2) together with the pre-F-containing core-shell implant on day 0. Mice in group C were immunized s.c. with pre-F (50 μg) diluted in PBS solution on day 0. Mice of group D were given a placebo core-shell implant s.c. on day 0. Mice of group E received 200 μL PBS solution s.c. on day 0 and week 3, as a negative control. Based on the previous study with OVA-PLGA core-shell implants [26], it can be expected that release from the core-shell implant starts 3–4 weeks after administration. Therefore, mice of group E were given a second PBS injection at week 3.

Mice of groups A, B, and D were surgically implanted with a core-shell implant. Briefly, the surgical procedure was as follows. Approximately 15 min before surgery, the analgesic Meloxicam (Metacam^®^, Boehringer Ingelheim, Germany, 5 mg/kg, s.c.) was administered, followed by isoflurane anaesthesia. The right abdominal side was shaved and disinfected with iso-Betadine^®^ and the core-shell implants were aseptically implanted. This was done by making a small incision at the right abdominal side and creating a s.c. pocket using sterile forceps and blunt-end scissors. The core-shell implants were placed on top of the s.c. musculature and the wound was closed by using surgical clips. After the procedure, the mice were placed under an infrared heating lamp to regain consciousness. Post-surgery, the mice were followed closely.

For sample collection, the mice were bled from the tail vein. This was done by making a small incision with a surgical blade. For each sample, an amount of 100 μL blood was collected in a Microvette^®^ CB 300Z clotting activator/serum tube. During blood sampling, mice were fully conscious and were gently restrained using a suitable restrainer. To obtain serum, the blood samples were incubated for at least 1 h at RT. After incubation, the samples were centrifuged for 4 min at 4000 rpm, followed by 1 min at 14,000 rpm at RT. The upper layer of transparent fluid (serum) was collected and transferred to a clean tube. Isolated serum was shielded from daylight and stored at −20 °C prior to analysis. Blood samples were taken 24 h after immunization and after weeks 1, 2, 3, 4, 6, 8, 10, and 12. In total, nine blood samples were taken from each mouse.

### 2.13. RSV VNA Titers

RSV VNA titers in mouse serum were measured by an automated microneutralization assay using RSV CL57V224-FFL grown on A549 cells. Serial diluted heat inactivated sera samples were mixed with 25,000 plaque-forming units (pfu) of RSV CL57V224-FFL in ½ area white tissue culture plates and incubated for 1 h at RT. Subsequently, 5 × 10^3^ A549 cells per well were added and the plates were incubated for 20 h at 37 °C and 10% CO_2_. After incubation, neolite substrate was added. The luminescence signal was determined with an EnVision plate reader (PerkinElmer, Waltham, MA, USA) set at enhanced luminescence mode. RSV VNA titers were calculated as the antibody concentration that caused a 90% reduction in luminescence, expressed as IC_90_ titers.

## 3. Results

### 3.1. Storage Stability of the Freeze-Dried Pre-F-Containing Powder: ELISA

**Figure 2:**
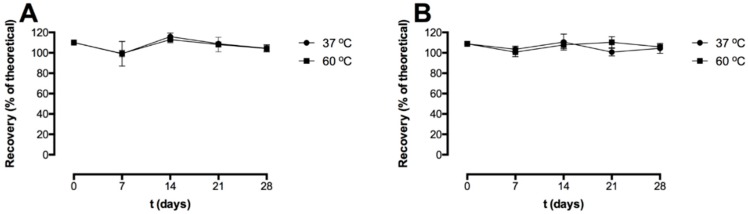
Recovery (% of theoretical) of the freeze-dried pre-F-containing powder immediately after freeze-drying and after storage at 37 °C (circle) and 60 °C (square). Two different detection antibodies were used; CR9501-biotin (**A**) and CR9506-biotin (**B**). The recovery was based on the theoretical pre-F content. For each time point, *n* = 3, mean and standard deviation are given.

Figure 2 shows the recovery after freeze-drying and the storage stability of the pre-F-containing powder formulation stored at 37 °C and 60 °C, as measured with capture ELISA. Two different detection antibodies were used; CR9501-biotin binds specifically to the pre-F conformation of the F protein (Figure 2A) and CR9506-biotin binds to both the pre-F conformation and the post-F conformation of the F protein (Figure 2B).

Immediately after freeze-drying (*t* = 0 days), the recovery with CR9501-biotin as detecting antibody was 110.1 ± 2.4%. The recovery immediately after freeze-drying (*t* = 0 days) with CR9506-biotin as detecting antibody was 108.8 ± 2.1%. After 28 days of storage at 37 °C and 60 °C, the recovery with CR9501-biotin as detecting antibody was 104.3 ± 3.5% and 104.1 ± 3.3%, respectively. The recovery with CR9506-biotin as detecting antibody after 28 days of storage at 37 °C and 60 °C was 104.4 ± 4.9% and 105.8 ± 2.0%, respectively.

### 3.2. Storage Stability of the Freeze-Dried Pre-F-Containing Powder: SEC

**Figure 3:**
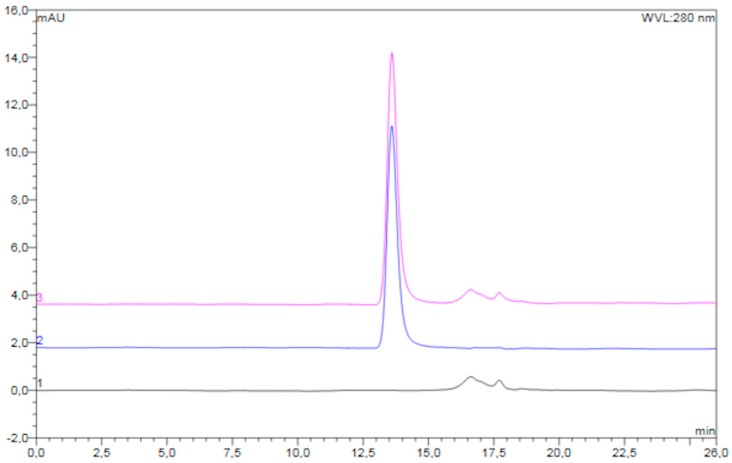
Overlay of typical chromatograms of the reconstituted freeze-dried placebo powder (black), unprocessed diluted pre-F (blue), and reconstituted freeze-dried pre-F-containing powder (pink).

An overlay of typical chromatograms of the reconstituted freeze-dried placebo powder, unprocessed diluted pre-F, and reconstituted freeze-dried pre-F-containing powder is shown in Figure 3.

The chromatogram of the reconstituted freeze-dried pre-F-containing powder (pink) showed a clear peak with a retention time of approximately 13.6 min. In addition, several smaller peaks were observed with retention times between 16 and 19 min. The chromatogram of unprocessed pre-F (blue) showed one clear peak with a retention time of approximately 13.6 min. The chromatogram of the reconstituted freeze-dried placebo powder (black) showed several smaller peaks, having retention times between 16 and 19 min.

**Figure 4:**
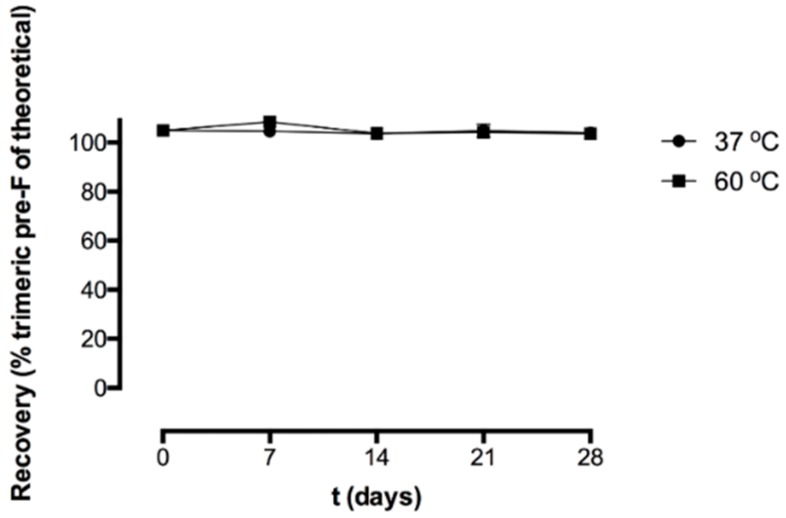
Recovery (% trimeric pre-F of theoretical) of the freeze-dried pre-F-containing powder formulation immediately after freeze-drying and after storage at 37 °C (circle) and 60 °C (square). For each time point, *n* = 3, mean and standard deviation are given.

Figure 4 shows the recovery of the freeze-dried pre-F-containing powder immediately after freeze-drying and after storage at 37 °C and 60 °C, as measured with SEC.

Immediately after freeze-drying (*t* = 0 days), the recovery of trimeric pre-F was 105.0 ± 1.2%. After 28 days of storage at 37 °C and 60 °C, the recovery of trimeric pre-F was 104.0 ± 0.2% and 103.7 ± 0.8%, respectively. Moreover, no additional peaks were observed in the chromatograms of reconstituted freeze-dried pre-F-containing powder.

### 3.3. Storage Stability of the Freeze-Dried Pre-F Containing Powder: Fluorescence Spectroscopy

**Figure 5:**
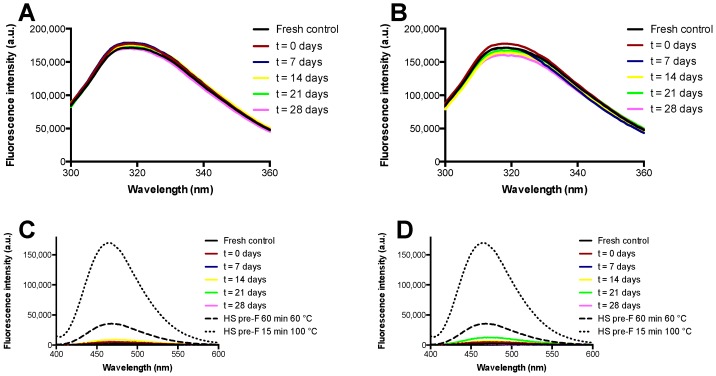
Stability of the freeze-dried pre-F-containing powder using fluorescence spectroscopy. (**A**) and (**B**) show the results of intrinsic fluorescence spectroscopy at storage temperatures of 37 °C and 60 °C, while (**C**) and (**D**) show the results of extrinsic fluorescence spectroscopy at storage temperatures of 37 °C and 60 °C. For each time point, the mean of three independent measurements is shown. Since the curves almost completely overlapped, error bars are not presented.

The results of the fluorescence spectroscopy study with the pre-F-containing powder immediately after freeze-drying and after storage are shown in Figure 5. Figure 5A,B show the results of intrinsic fluorescence spectroscopy after storage at temperatures of 37 °C and 60 °C, while Figure 5C,D show the results of extrinsic fluorescence spectroscopy after storage at temperatures of 37 °C and 60 °C. Furthermore, these figures show the spectra of heat shocked samples of pre-F.

Intrinsic fluorescence spectroscopy showed that unprocessed diluted pre-F excited at a wavelength of 295 nm had an emission maximum at approximately 318 nm. The spectra of the reconstituted freeze-dried pre-F-containing powder after freeze-drying and all spectra of the powder stored at 37 °C and 60 °C overlapped with the spectrum obtained from unprocessed diluted pre-F.

Extrinsic fluorescence spectroscopy showed that all the reconstituted freeze-dried pre-F-containing samples had low fluorescence intensities and overall fluorescence spectra similar to that of unprocessed diluted pre-F. As can be seen, heat shocking unprocessed diluted pre-F for 60 min at 60 °C resulted in a clear increase in fluorescence intensity in combination with a blueshift of the emission maximum. This effect was even more pronounced when unprocessed diluted pre-F was exposed to a heat shock for 15 min at 100 °C.

### 3.4. Influence of the Core-Shell Production Process on the Stability of Pre-F

**Figure 6:**
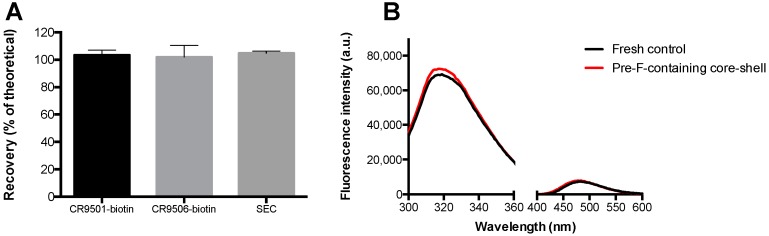
Analyses on the pre-F-containing core-shell implants to investigate the influence of the core-shell production process on the stability of pre-F. (**A**) shows the recovery (% of theoretical) of the pre-F-containing core-shell implants found with ELISA (CR9501-biotin (black bar) and CR9506-biotin (light grey bar) as detection antibodies) and SEC (dark grey bar). For each analytical technique, *n* = 3, mean and standard deviation are given. (**B**) shows the results of intrinsic (300–360 nm) and extrinsic (400–600 nm) fluorescence spectroscopy of the pre-F-containing core-shell implants (red line) compared to a fresh unprocessed control with the same theoretical concentration (black line). For each group, the mean of three independent measurements is shown. Since the curves almost completely overlapped, error bars are not presented.

The results of the analyses performed on the pre-F-containing core-shell implants to investigate the influence of the core-shell production process on the stability of pre-F are shown in Figure 6. Figure 6A shows the results of ELISA (CR9501-biotin and CR9506-biotin as detection antibodies, respectively) and SEC. Figure 6B shows the results of fluorescence spectroscopy (intrinsic fluorescence spectroscopy (300–360 nm) and extrinsic fluorescence spectroscopy (400–600 nm)).

After incorporation into core-shell implants, recoveries of 103.4 ± 3.6%, 102.0 ± 8.5%, and 104.7 ± 1.7% were found with CR9501-biotin, CR9506-biotin, and SEC, respectively. The overall fluorescence spectra obtained from pre-F-containing core-shell implants were similar to those of a fresh unprocessed pre-F control with the same theoretical concentration.

### 3.5. In Vitro Release Study with Pre-F-Containing Core-Shell Implants

**Figure 7:**
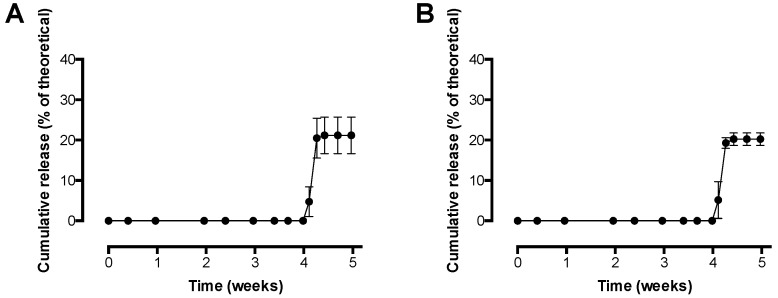
In vitro release of pre-F from pre-F-containing core-shell implants. (**A**) shows the release of pre-F measured with CR9501-biotin as detection antibody, while (**B**) shows the release of pre-F measured with CR9506-biotin as detection antibody. For each time point, *n* = 3, mean and standard deviation are given.

Figure 7 shows the results of the in vitro release study with pre-F-containing core-shell implants. Figure 7A shows the release of pre-F from the core-shell implants measured with CR9501-biotin as detection antibody, while Figure 7B shows the release of pre-F from the core-shell implants measured with CR9506-biotin as detection antibody.

The results of the in vitro release study with pre-F-containing core-shell implants showed that release from the core-shell implants was measured after a lag time of 4 weeks with both detection antibodies. After approximately 4.5 weeks, an incomplete maximal cumulative release of 21.2 ± 4.5% and 20.3 ± 1.6% was measured with CR9501-biotin and CR9506-biotin, respectively.

### 3.6. In Vivo Study with Pre-F-Containing Core-Shell Implants

**Figure 8:**
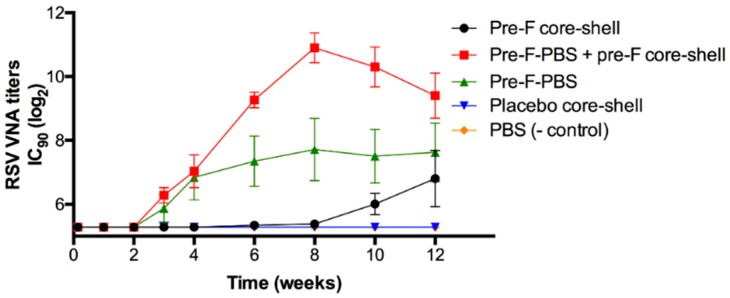
RSV VNA titers over time. Pre-F core-shell = black circle, pre-F-containing core-shell implant at day 0; Pre-F-PBS + pre-F core-shell = red square, s.c. injection pre-F at day 0 and pre-F-containing core-shell implant at day 0; Pre-F-PBS = green triangle, s.c. pre-F injection at day 0; Placebo core-shell = blue triangle, placebo core-shell implant at day 0; PBS (- control) = orange diamond, s.c. injection PBS at day 0 and s.c. injection PBS at week 3. Each group contained 6 mice, except PBS (- control), which contained 3 mice. Mean RSV VNA titers and standard error of mean are presented.

Figure 8 shows the RSV VNA titers over time for the in vivo study with pre-F-containing core-shell implants.

Approximately 3 weeks after s.c. immunization with an injection of pre-F (green triangle), RSV VNA titers were detected. The titers reached a plateau after approximately 8 weeks. Mice to which a pre-F-containing core-shell implant was administered (black circle), showed a delayed RSV VNA response compared to mice that were immunized with an injection of pre-F, i.e., after week 8, RSV VNA titers were detectable. This lag time can be ascribed to the combined effect of the delayed release of the antigen (approximately 4 weeks) and the time between release and detection of RSV VNA titers in serum (approximately 3 weeks). After 12 weeks, RSV VNA titers of mice immunized with a s.c. pre-F injection and mice immunized with a pre-F-containing core-shell implant were comparable (log_2_IC_90_ = 7.6 ± 0.9 and 6.8 ± 0.9, respectively). To mimic a single-injection system containing a primer and a booster, mice received a s.c. injection of pre-F (primer) in combination with a s.c. administered pre-F-containing core-shell implant (booster). In the figure, this group is depicted by the red line with red squares. For this group, RSV VNA titers continued to increase after 4 weeks and higher RSV VNA titers were observed compared to mice immunized with a s.c. pre-F injection or compared to mice that were only administered a pre-F-containing core-shell implant. After 8 weeks, RSV VNA titers declined but remained higher than RSV VNA titers of mice that received only one immunization (Pre-F core-shell and Pre-F-PBS). As expected, no RSV VNA titers were detected in mice that received a placebo core-shell implant (blue triangle) and mice that received a prime-boost injection of PBS (orange diamond).

## 4. Discussion

To increase the flexibility regarding the route of administration of a potential pre-F vaccine, we sought to develop a stable freeze-dried pre-F-containing powder. The conformational integrity of the protein in the freeze-dried powder was investigated with ELISA by using CR9506 as capturing antibody in combination with either CR9501-biotin or CR9506-biotin as the detection antibody. CR9501-biotin binds specifically to the pre-F conformation but not to the post-F conformation. The other detection antibody, CR9506-biotin, binds to the pre-F conformation as well as the post-F conformation. As the capturing antibody (CR9506) binds to the same epitope as CR9506-biotin, CR9506-biotin cannot bind to monomeric F protein. The ELISA showed that pre-F did not lose its conformation during freeze-drying. Moreover, as similar recoveries were found with CR9501-biotin and CR9506-biotin, the results indicate that all F protein was in the pre-F conformation and was di- and/or trimeric immediately after freeze-drying. As similar results were found after 28 days of storage, the F protein remained in the di- and/or trimeric pre-F conformation for at least 28 days at 37 °C and 60 °C.

To investigate whether irreversible soluble aggregates and/or fragments were present and to quantify trimeric pre-F, SEC was applied. The clear peak with a retention time of 13.6 min in the chromatogram of reconstituted freeze-dried pre-F-containing powder can be ascribed to trimeric pre-F, as unprocessed diluted pre-F also showed this peak. The additional smaller peaks observed in the chromatogram of reconstituted freeze-dried pre-F-containing powder can be ascribed to the excipients present, as these peaks were also observed in the chromatogram of reconstituted freeze-dried placebo powder. Since the peaks of the excipients are clearly separated from the trimeric pre-F peak, SEC can be used to quantify trimeric pre-F. SEC showed that pre-F remained in the trimeric conformation during freeze-drying. The results also showed that pre-F did not lose its trimeric conformation up to 28 days of storage at temperatures of 37 °C and 60 °C. Moreover, since no additional peaks were observed in the chromatograms of the reconstituted freeze-dried pre-F-containing powder, irreversible soluble aggregates and/or fragments were absent [27]. These results confirm the results found with ELISA.

For intrinsic fluorescence spectroscopy, the samples were excited at a wavelength of 295 nm to selectively excite tryptophan (Trp) residues of the protein [28]. The fluorescence spectrum of Trp is greatly dependent on its local environment [28,29]. Generally, a blueshift is observed with decreasing solvent polarity, for instance, with protein aggregation when the Trp residues are buried [28,29]. On the other hand, a redshift is observed when the Trp residues are solvent-exposed, for instance with protein denaturation [29]. Moreover, the fluorescence intensity of Trp is influenced by its local environment [29]. As no shifts in emission maxima and clear differences in fluorescence intensities were observed, pre-F maintained its native conformation up to 28 days of storage at temperatures of 37 °C and 60 °C. These results are in line with the results obtained with ELISA and SEC.

For the extrinsic fluorescence spectroscopy, ANS was used as fluorescent dye. The fluorescence of ANS is greatly dependent on the polarity of its environment [28]. In an aqueous environment, ANS is hardly fluorescent [28,30]. However, the fluorescence of ANS increases significantly in a less polar environment, for example in the hydrophobic pockets of protein aggregates [28,30]. Moreover, a blueshift of the emission maximum is observed in a less polar environment [28,30]. The heat shocked samples investigated in this study showed an increase in fluorescence intensity and a blueshift, indicating that heat shocking indeed resulted in protein degradation. Extrinsic fluorescence spectroscopy is of interest because it is known that already low concentrations of aggregates can be detected with this technique [28]. The reconstituted freeze-dried pre-F-containing samples all showed low fluorescence intensities and fluorescence spectra similar to that of the fresh control. Therefore, extrinsic fluorescence spectroscopy also indicates that the freeze-dried pre-F-containing powder maintains its native conformation, which is in line with the results found with ELISA, SEC, and intrinsic fluorescence spectroscopy.

Overall, the results showed that the developed formulation, consisting of pre-F protein incorporated in a stabilizing matrix containing inulin (1:99 *w*/*w*), 20 mM HEPES (pH 7.5), 65 mM NaCl, and 0.02% (*v*/*v*) Tween 80, was stable during freeze-drying and subsequent storage for at least 28 days at 37 °C and 60 °C. Because the pre-F conformation is known to more effectively induce RSV neutralizing responses compared to the post-F conformation [11,12], it is important for a pre-F-based RSV subunit vaccine that the F protein remains in the pre-F conformation upon drying and subsequent storage, which was the case for the developed freeze-dried pre-F-containing powder. Our results are in line with other studies showing that inulin can be used to stabilize proteins during freeze-drying and subsequent storage [31]. In addition, the other excipients, HEPES [32], surfactants (e.g., Tween 80), and salts [17,18], have been described as protein stabilizers during freeze-drying and/or subsequent storage.

Since the antigen is in the dry state, it can easily be incorporated into several other dosage forms, amongst which a solid state single-injection system containing a primer and a booster dose of pre-F. This dosage form is particularly challenging because it should release the primer dose immediately after administration, followed by the release of the booster after a specific lag time. During this lag time, the antigen is exposed to an elevated temperature, i.e., body temperature. Since the pre-F-containing powder was found to be stable during prolonged storage at 37 °C and 60 °C, we investigated whether incorporation of the developed freeze-dried pre-F-containing powder into a core-shell implant is feasible and whether this system induces a delayed RSV VNA response in mice. In addition, we investigated whether the anticipated concept of a primer and booster administration in one injection could be feasible and indeed would induce an increased immune response.

Incorporation of the pre-F-containing powder into the core-shell implant was feasible and the core-shell production process did not affect the stability of pre-F (based on ELISA, SEC, and fluorescence spectroscopy). An in vitro release study showed that pre-F was released from the core-shell implant after a lag time of 4 weeks, which is in line with the previous study where OVA was released from the core-shell implant after 3–4 weeks [26]. The observed delayed release can be ascribed to the fact that pre-F is not immediately released from the nonporous core-shell implant. Before release can take place, the polymer has to be degraded to such an extent that it can no longer serve as a barrier [26]. Since the releases measured with CR9501-biotin and CR9506-biotin were similar, all measured F protein was in the pre-F conformation and was di- and/or trimeric. However, an incomplete pre-F release was observed. Such an incomplete protein release from PLGA-based implants, ascribed to protein instability within the implant, has often been described in literature, even for relatively stable model proteins (e.g., BSA and OVA) [33,34,35]. Important factors contributing to protein instability within PLGA-based implants are the development of an acidic microclimate within the matrix and incompatibility of the protein with PLGA degradation products [36,37,38]. To overcome these issues, and thereby potentially increasing protein release, additional excipients (e.g., shellac and magnesium hydroxide) could be incorporated into the formulation [35,39,40]. The aforementioned factors may also have led to the incomplete release of pre-F from the core-shell implant, however, further research is needed to elucidate this and to investigate whether these issues may be overcome by incorporating additional excipients. Since the current core-shell implant did however show the desired delayed release of approximately 10 μg di- and/or trimeric pre-F in vitro, the system was evaluated in vivo as proof of concept.

In vivo, approximately 3 weeks after injection, RSV VNA titers were detected in mice that were s.c. immunized with pre-F at day 0. This indicates that it takes approximately 3–4 weeks after administration of the antigen before RSV VNA titers can be detected in mice. The much longer delay in RSV VNA response observed after administration of a pre-F-containing core-shell implant can be ascribed to the fact that pre-F is not immediately released from the nonporous core-shell implant but after a lag time of approximately 4 weeks, which is in line with the results of the in vitro release study. Since it takes another 3–4 weeks after antigen release before RSV VNA titers can be detected in mice, depending on the vaccine modality, it can be expected that RSV VNA titers in mice immunized with a pre-F-containing core-shell implant can be detected after week 8, which indeed was the case. These results suggest that incorporation of the developed freeze-dried pre-F-containing powder into core-shell implants is suitable to induce a delayed RSV VNA response compared to a s.c. injection of pre-F. To mimic a single-injection vaccine containing a primer and a booster, mice were primed with a s.c. injection of pre-F in combination with a s.c. administered pre-F-containing core-shell implant. Up to 4 weeks, no differences were observed in the RSV VNA titers of mice immunized with a s.c. injection of pre-F in combination with a s.c. administered pre-F-containing core-shell implant and mice immunized with a single s.c. injection of pre-F. These results indicate that PLGA does not have an adjuvant effect on the RSV VNA titers. After 4 weeks, the release of pre-F from the core-shell implant immediately increased the RSV VNA titers because the mice were primed. Therefore, the 3–4 weeks between antigen release and detection of RSV VNA titers no longer occurred. As a result, the highest RSV VNA titers were found for this group. Overall, these results show that incorporation of the developed freeze-dried pre-F-containing powder into core-shell implants is suitable to boost the RSV VNA response. These results are in line with the previous study with OVA-PLGA core-shell implants [26].

As the described core-shell implant is obviously too big for clinical application, miniaturization of the device is necessary. In addition, miniaturization may be beneficial to the protein stability within the implant during the lag time, as it would result in an implant with significant less PLGA around the core than the current core-shell implant. One way to achieve miniaturization may be by using commercially available hollow nonporous PLGA tubes. In a preliminary study, we investigated the suitability of such a tube to obtain a delayed release. The tubes used for this preliminary study were commercially available PLGA 50:50 tubes (Zeus Industrial Products, Inc., Orangeburg, SC, USA) with an inner diameter (I.D.) of approximately 500 μm, a wall thickness of approximately 100 μm, and therefore an outer diameter of approximately 700 μm (Figure S1). With these dimensions, the tube would fit into an 18G injection needle (I.D. of 838 μm), making it clinically applicable. In the preliminary study, the tubes were first closed at one end by slightly dissolving a piece of PLGA material in a few drops of acetone and using the resulting viscous mixture to manually form a plug on the PLGA tube. The tubes were then filled with aminophylline powder (model drug) by simply dipping the open end of the tube in the powder, shoving the powder with a thin-walled 27G needle, and briefly centrifuging the tubes containing powder. After filling, the other end of the tube was closed as described above. A subsequent in vitro release study of these tubes showed a delayed (approximately 3–4 weeks) release of aminophylline from the PLGA tubes (Figure S2). These preliminary results suggest that hollow nonporous PLGA tubes may indeed be suitable to miniaturize the core-shell implant described in this paper. The primer could possibly be included by providing the outer surface of the tube with an additional layer of powder. Finally, this may result in a clinically applicable single-injection vaccine containing a primer and a booster.

## 5. Conclusions

The developed freeze-dried powder formulation consisted of pre-F incorporated in a stabilizing matrix of inulin, HEPES (pH 7.5), sodium chloride, and Tween 80. In this formulation, the protein was stable during freeze-drying and subsequent storage for at least 28 days at temperatures of 37 °C and 60 °C. The antigen did not lose the pre-F conformation, as confirmed with ELISA. SEC showed that pre-F maintained trimeric and showed the absence of irreversible soluble aggregates and/or fragments. The results of fluorescence spectroscopy (intrinsic and extrinsic) showed that pre-F maintained its native conformation. Compared to a liquid pre-F vaccine formulation, the developed freeze-dried powder has an increased flexibility regarding the route of administration and the dosage form. Incorporation of the developed freeze-dried pre-F-containing powder into a core encapsulated by a nonporous PLGA shell was feasible and did not affect the stability of pre-F. An in vitro release study with pre-F-containing core-shell implants showed that di- and/or trimeric pre-F was incompletely released from the core-shell implants after a lag time of 4 weeks. The incomplete release may be the result of pre-F instability within the core-shell implant during the lag time and requires further research. The in vivo study showed that subcutaneous administration of pre-F-containing core-shell implants resulted in a delayed RSV VNA response that corresponded with pre-F release from the core-shell implant after a lag time of approximately 4 weeks. In addition, pre-F-containing core-shell implants were able to boost RSV VNA titers of primed mice after a lag time of 4 weeks, demonstrating the feasibility of the biphasic pulsatile release profile. The findings described in this paper could contribute to the development of a pre-F-based vaccine and to the development of a single-injection vaccine containing a primer and a booster.

## Figures and Tables

**Table 1:** Overview of the groups and formulations used for the in vivo study.

Group	Formulation	Day 0	Week 3
A	Pre-F core-shell implant	Pre-F core-shell implant	-
B	Pre-F-PBS + pre-F core-shell implant	Pre-F-PBS + pre-F core-shell implant	-
C	Pre-F-PBS	Pre-F-PBS	-
D	Placebo core-shell implant	Placebo core-shell implant	-
E	PBS (- control)	PBS	PBS

## Supplementary Materials

The following are available online at https://www.mdpi.com/1999-4923/11/10/510/s1. Figure S1: Scanning electron microscopy image (top view) of a commercially available hollow nonporous PLGA 50:50 tube at a magnification of 100×. The tube has an inner diameter of approximately 500 μm, a wall thickness of approximately 100 μm, and therefore an outer diameter of approximately 700 μm. Figure S2: In vitro release of aminophylline from PLGA tubes. Standard deviation is indicated (*n* = 3).
