# Supplementary material for: Development of a Stable Respiratory Syncytial Virus Pre-Fusion Protein Powder Suitable for a Core-Shell Implant with a Delayed Release in Mice: A Proof of Concept Study
Source: Pharmaceutics. 2019 Oct 3;11(10):510. doi: 10.3390/pharmaceutics11100510 (PMC6835792; doi:10.3390/pharmaceutics11100510)
Supplement: Supplementary file 1 [file pharmaceutics-11-00510-s001.docx]

Supplementary Materials: Development of a Stable Respiratory Syncytial Virus Pre-Fusion Protein Powder Suitable for a Core-Shell Implant with a Delayed Release in Mice: A Proof of Concept Study

Max Beugeling, Katie Amssoms, Freek Cox, Ben De Clerck, Ellen Van Gulck,
Jeroen A. Verwoerd, Guenter Kraus, Dirk Roymans, Lieven Baert, Henderik W. Frijlink and Wouter L. J. Hinrichs


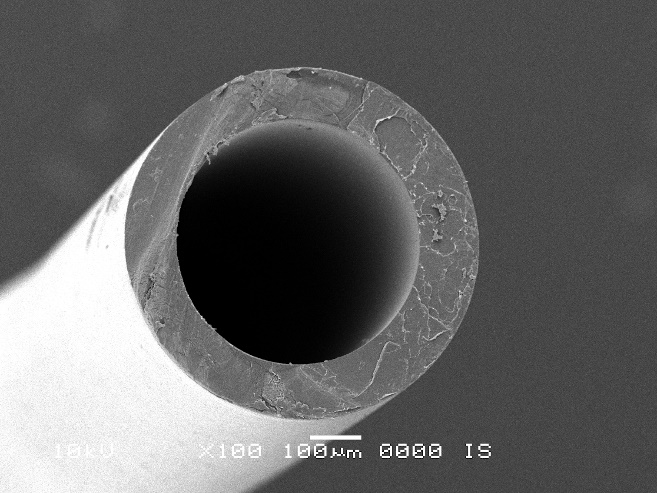


**Figure S1.** Scanning electron microscopy image (top view) of a commercially available hollow nonporous PLGA 50:50 tube at a magnification of 100×. The tube has an inner diameter of approximately 500 μm, a wall thickness of approximately 100 μm, and therefore an outer diameter of approximately 700 μm.


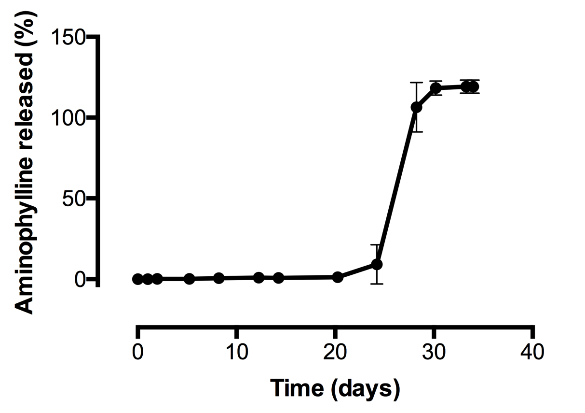


**Figure S2.** In vitro release of aminophylline from PLGA tubes. Standard deviation is indicated (*n* = 3).
